# Supplementary material for: GALNTL5 binds GalNAc and is required for migration through the uterotubal junction and sperm-zona pellucida binding
Source: Nat Commun. 2025 Sep 17;16:8264. doi: 10.1038/s41467-025-63805-4 (PMC12443989; doi:10.1038/s41467-025-63805-4)
Supplement: Supplementary file 1 — Supplementary Information [file 41467_2025_63805_MOESM1_ESM.pdf]

Supplementary Information
of
GALNTL5 binds GalNAc and is required for migration through the uterotubal junction and sperm-zona pellucida binding

Taichi Noda[1,2,3,#,*], Reika Uriu[1,#], Daisuke Mashiko[3], Hina Shinohara[1], Yongcun Qu[4], Ayumu Taira[1], Ryan M. Matzuk[5], Duri Tahala[1], Motochika Nakano[1], Kimi Araki[1,6], Zhifeng Yu[5], Ying Zhang[7], Martin M. Matzuk[5,*], and Masahito Ikawa[3,8,9,10,*]

[1]Institute of Resource Development and Analysis, Kumamoto University, 2-2-1 Honjo, Chuo-ku, Kumamoto, Kumamoto 860-0811, Japan
[2]Priority Organization for Innovation and Excellence, Kumamoto University, 2-39-1 Kurokami, Chuo-ku, Kumamoto, Kumamoto 860-8555, Japan
[3]Research Institute for Microbial Diseases, The University of Osaka, 3-1 Yamadaoka, Suita, Osaka 565-0871, Japan
[4]Institute of Artificial Intelligence in Sports (IAIS), Capital University of Physical Education and Sports, Beijing 100000, P R. China
[5]Center for Drug Discovery and Department of Pathology & Immunology, Baylor College of Medicine, One Baylor Plaza, Houston, TX 77030, USA
[6]Center for Metabolic Regulation of Healthy Aging, Kumamoto University, 1-1-1, Honjo, Chuo-ku, Kumamoto, Kumamoto 860-8556, Japan
[7]The Key Laboratory of Cell Proliferation and Regulation Biology, Ministry of Education, College of Life Sciences, Beijing Normal University, Beijing, China, 100875.
[8]The Institute of Medical Science, The University of Tokyo, 4-6-1 Shirokanedai, Minato-ku, Tokyo 108-8639, Japan
[9]Center for Infectious Disease Education and Research (CiDER), The University of Osaka, Suita, Osaka 565-0871, JAPAN.
[10]Center for Advanced Modalities and DDS (CAMaD), The University of Osaka, Suita, Osaka 565-0871, JAPAN.
[#]Both authors equally contributed to this work.
[*]Correspondences and requests for materials should be addressed to T.N. (email: noda-t@kumamoto-u.ac.jp), M.M.M. (email: mmatzuk@bcm.edu), or M.I. (email: ikawa@biken.osaka-u.ac.jp)

This file contains:
Supplementary Figures 1-8
Supplementary Tables 1-4
Supplementary References

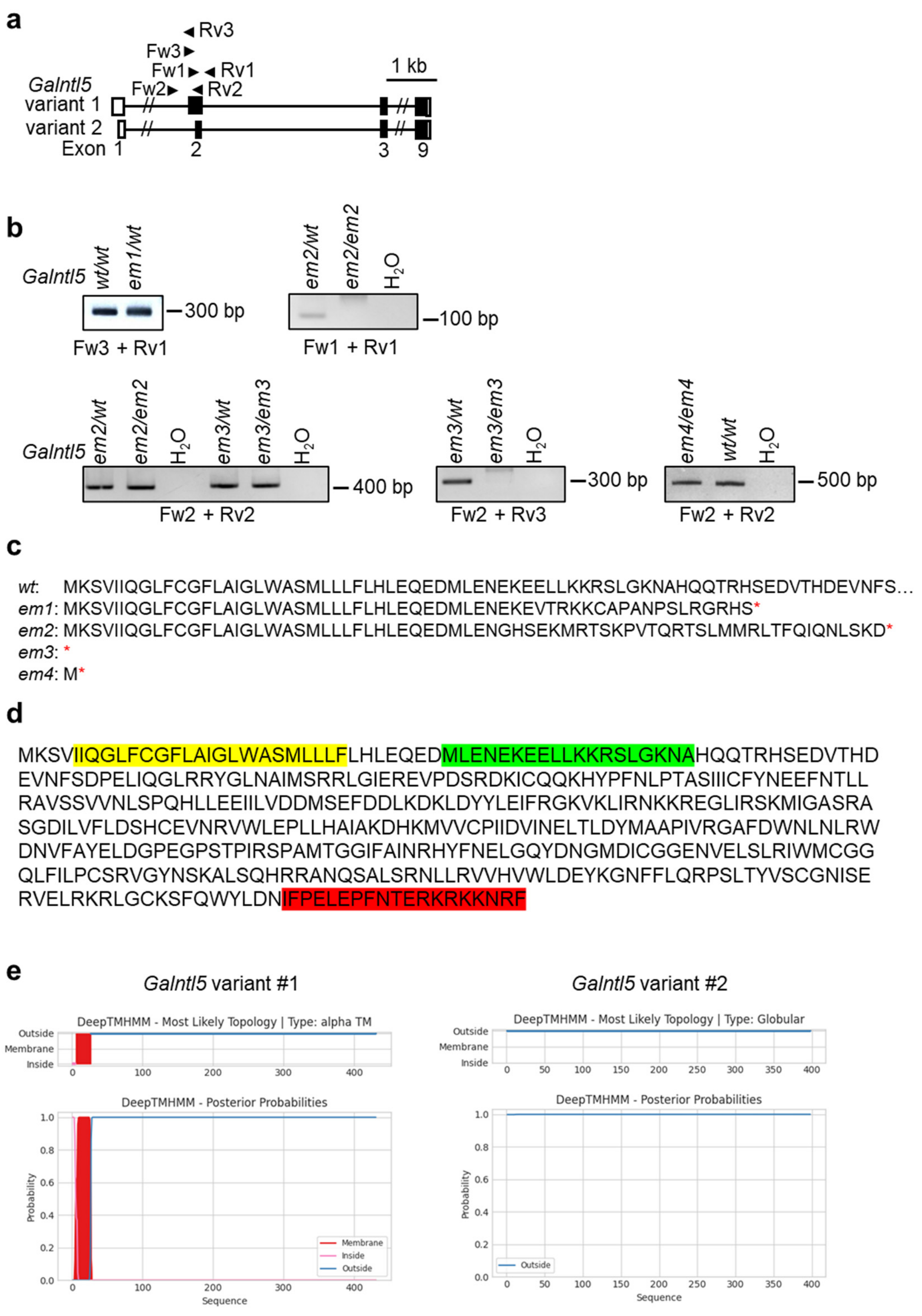

Supplementary Figure 1. Genotyping PCR to detect *Galntl5* mutants and the topology of GALNTL5 proteins.

a) **Primer design.** The detailed sequence of each primer is shown in Supplementary Table 2. Fw: forward, Rv: reverse.

b) Genotyping PCR. Primers in panel a were used for PCR.

c) Predicted amino acid sequence. The *em1* and *em2* alleles have 17 and 25 nucleotide (nt) deletions, respectively (also see Fig. 1e). The *em3* and *em4* alleles have the indel mutation (8 nt deletion and 3 nt insertion) and 2 nt insertion, respectively (also see Fig. 5b). Thus, the premature stop codon (red asterisks) appears in proteins encoded by the transcripts from mutant alleles.

d) Antigen sites to generate anti-mouse GALNTL5 antibodies. The amino acid sequence translated from *Galntl5* variant 1 mRNA is shown (UniProt ID: Q9D4M9). Yellow: Predicted transmembrane domain, Green: Antigen sequence for a GALNTL5 (N) antibody, Red: Antigen sequence for a GALNTL5 (C) antibody. The detailed information is also shown in Supplemental Table 4.

e) TMHMM analysis. The topology of proteins encoded by variants 1 and 2 was analyzed through Deep TMHMM.

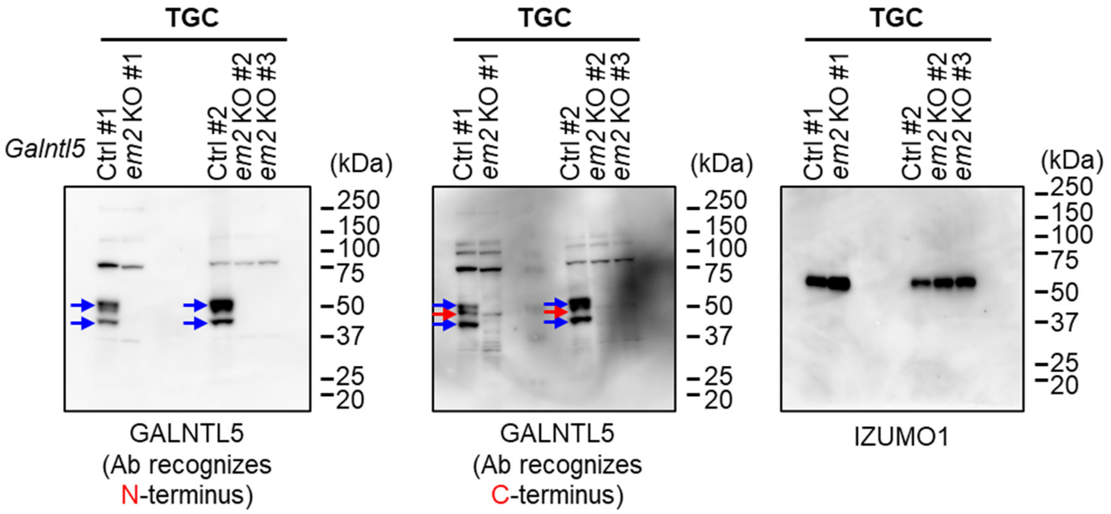

Supplementary Figure 2. Detection of GALNTL5 proteins in the TGC. Two control (#1-2) and three *Galntl5*^{em2} KO (KO #1-3) males were used to collect the TGC. TGC lysates (100 µg) were used for SDS-PAGE. Testicular GALNTL5 was detected using the N-terminus antibody, and then the membrane, after stripping the N-terminus antibody, was re-probed with the C-terminus antibody. Blue and red arrows show immature forms and a non-specific band, respectively. IZUMO1 was used for loading control.

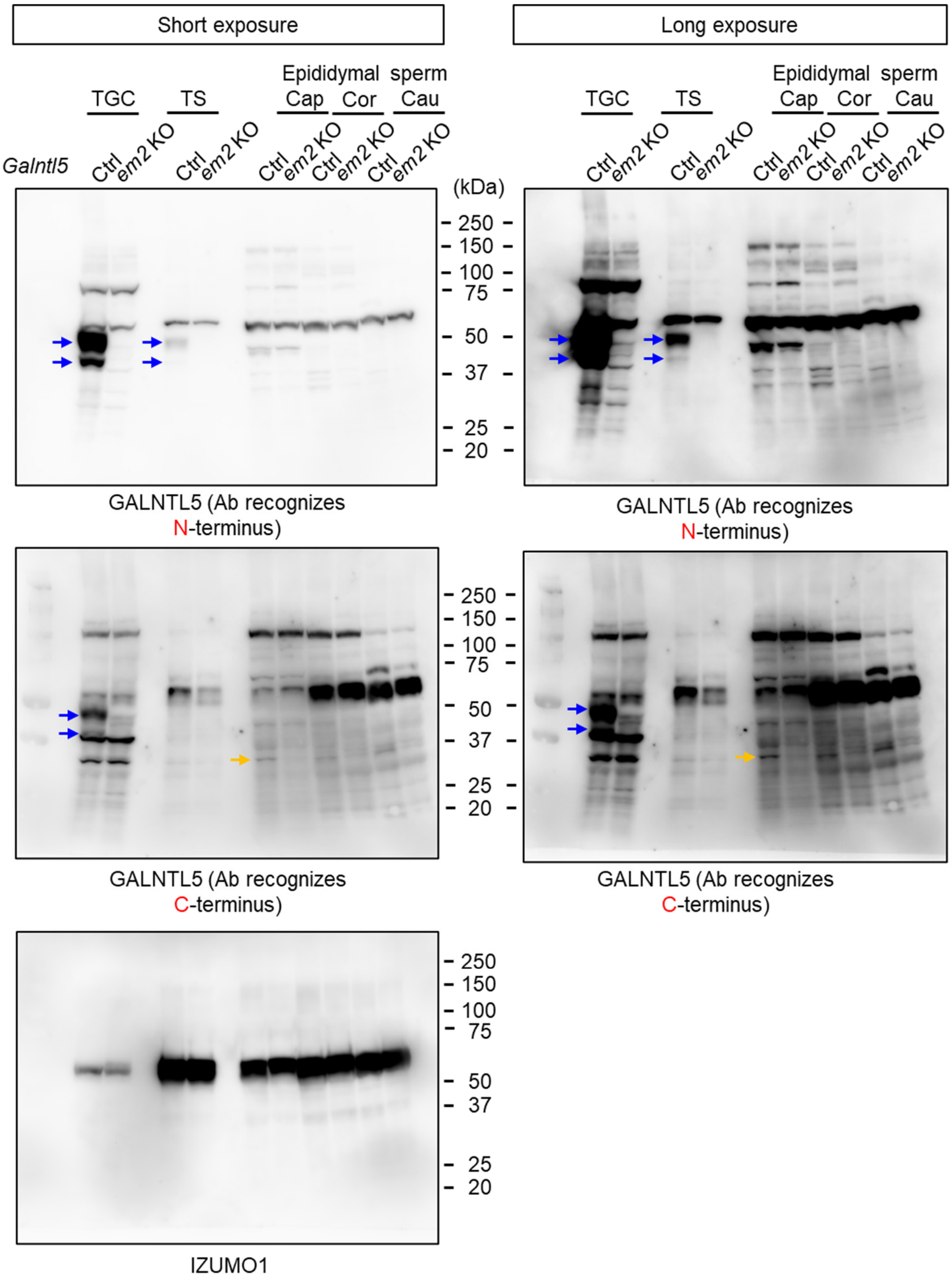

Supplementary Figure 3. Detection of GALNTL5 in TGC, TS, and epididymal sperm. TGC (100 µg), TS (40 µg), and caput (Cap, 110 µg), corpus (Cor, 110 µg) and cauda (Cau, 50 µg) epididymal sperm were used for SDS-PAGE. For the immunoblot analysis of GALNTL5, we used two antibodies to recognize the N- and C-termini of mouse GALNTL5 (see Fig. 1f and Supplementary Fig. 1d). We labeled immature (~50

kDa and ~46 kDa) and mature (~37 kDa) forms with blue and yellow arrows, respectively. Using an N-terminus antibody, the immature forms were not detected in the epididymal sperm but in TGC and TS. Using a C-terminus antibody, we barely found any immature forms in TS due to the poor reactivity of this antibody. However, the immature forms were detected in TGC, and the mature form could be detected in epididymal sperm after the caput region. IZUMO1 was used as the loading control. The data reproducibility was checked by two biological replicates.

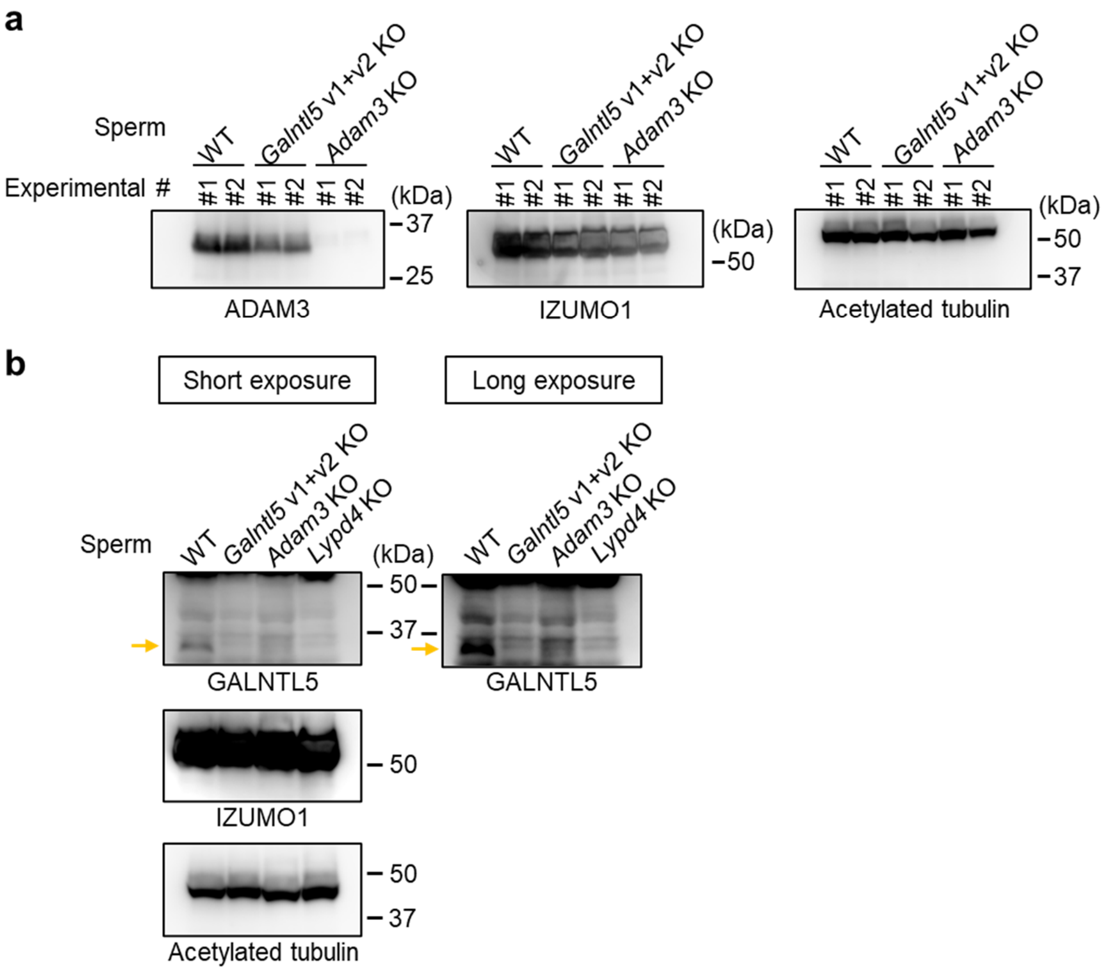

Supplementary Figure 4. Detection of ADAM3 and GALNTL5 in sperm lacking UTJ migration-related genes.

a) Detection of sperm ADAM3 in *Galntl5* KO sperm. The signal intensity of ADAM3 is reduced in *Galntl5* KO sperm, but ADAM3 remains (also see Fig. 4b). IZUMO1 and acetylated tubulin were used for loading controls. The data reproducibility was checked by five biological replicates.

b) Detection of GALNTL5 in *Adam3* KO and *Lypd4* KO sperm. The mature form of GALNTL5 was detected at ~37 kDa (yellow arrow), but the GALNTL5 signal almost disappears in *Adam3* KO and *Lypd4* KO sperm (see also Fig. 4f). The data reproducibility was checked by five biological replicates.

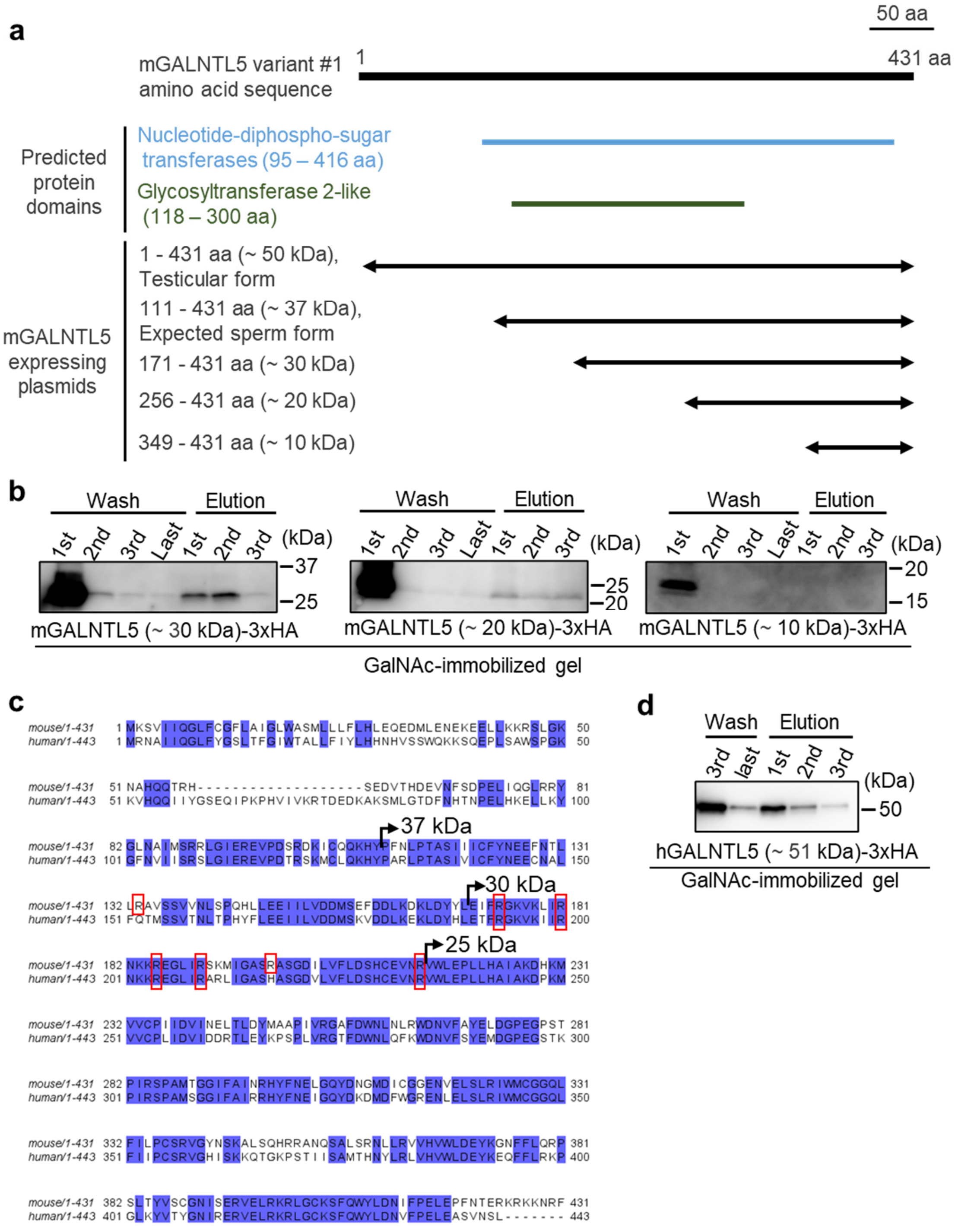

Supplementary Figure 5. Identification of the GalNAc binding region in GALNTL5.

a) **Plasmid construction.** Five expression vectors with amino acids of about 10, 20, 30, 37, and 50 kDa from the C-terminal of GALNTL5 were generated.

b) **GalNAc-binding ability of mouse GALNTL5 proteins.** GALNTL5 proteins were incubated in the GalNAc-immobilized gels (see Fig. 6c), and the obtained washed and elution buffers were used for western blot analysis. GALNTL5 proteins with

more than 20 kDa containing the predicted glycosyltransferase 2-like domain could be detected in the elution buffer. The data reproducibility was checked by two biological replicates.

c) **Comparison of amino acid sequences of mouse and human GALNTL5.** Blue colored letters show the matched amino acids between mice and humans. Based on the western blot data using testicular germ cells and sperm (see Fig. 1f and Supplementary Fig. 3), we speculate that GALNTL5 is cleaved between 37 kDa and 25 kDa from the C-terminal of GALNTL5 by some proteases. The serine protease "OVCH2", which exists in the caput epididymis and is essential for sperm migration through the UTJ, recognizes arginine (R) for the cleavage of target proteins. Thus, arginine was shown by the red squares.

d) **GalNAc-binding ability of human GALNTL5.** After incubating human GALNTL5 proteins in the GalNAc-immobilized gel, human GALNTL5 could be detected in the elution buffer. The data reproducibility was checked by two biological replicates.

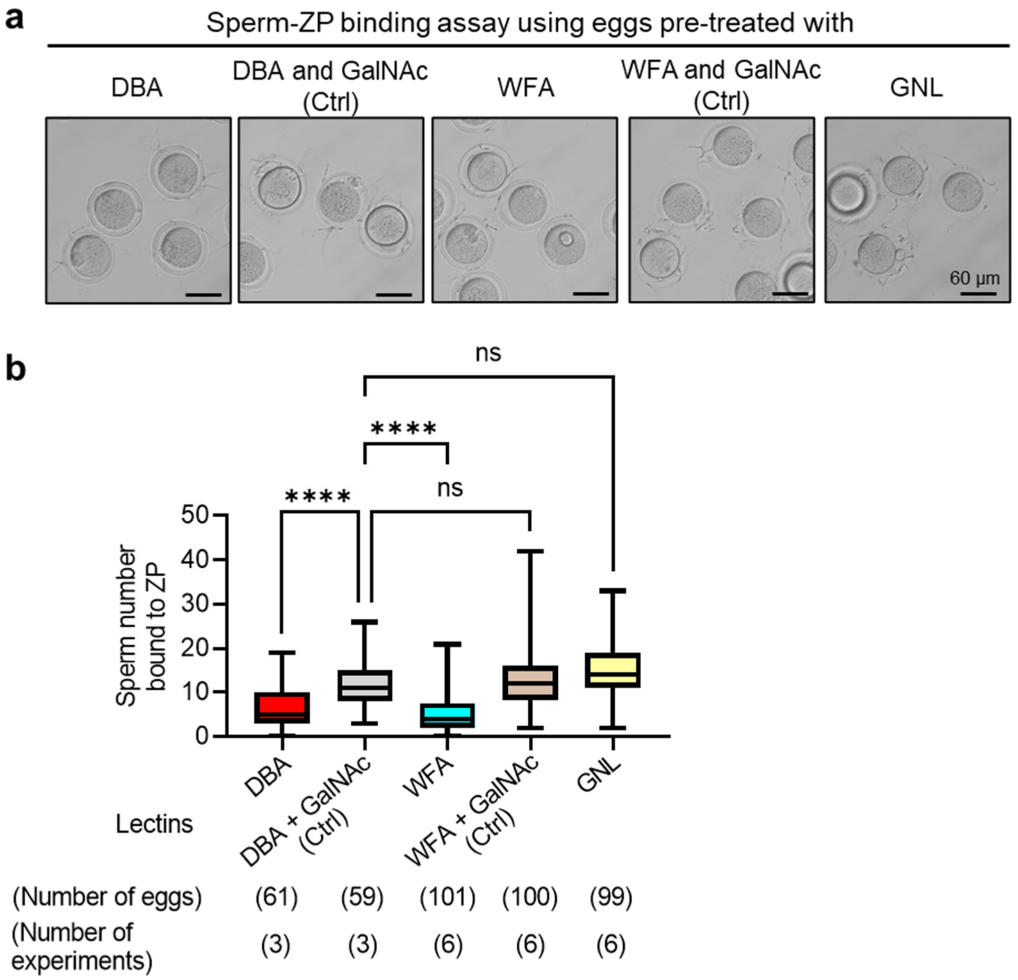

Supplementary Figure 6. Sperm-ZP binding assay by blockage of GalNAc and mannose on the ZP surface. DBA, WFA, and GNL bind to terminal α-GalNAc, GalNAc, and α-mannose residues, respectively. DBA and WFA pre-absorbed GalNAc were used as the control (Ctrl). Eggs were pre-incubated in TYH drop with each lectin, washed, and then inseminated with sperm. After a 10-minute incubation, eggs were observed (panel a) and the sperm bound to the ZP were counted (panel b) (DBA: 6.1 ± 4.4, DBA + GalNAc: 12.1 ± 5.2, WFA: 5.8 ± 5.2, WFA + GalNAc: 12.8 ± 6.6, GNL: 14.6 ± 7.0) (Kruskal-Wallis test, $p < 0.0001$). Center line within the box: median, whiskers in box-and-whiskers plots: minimum to maximum, ns: not significant, ****: $p < 0.0001$.

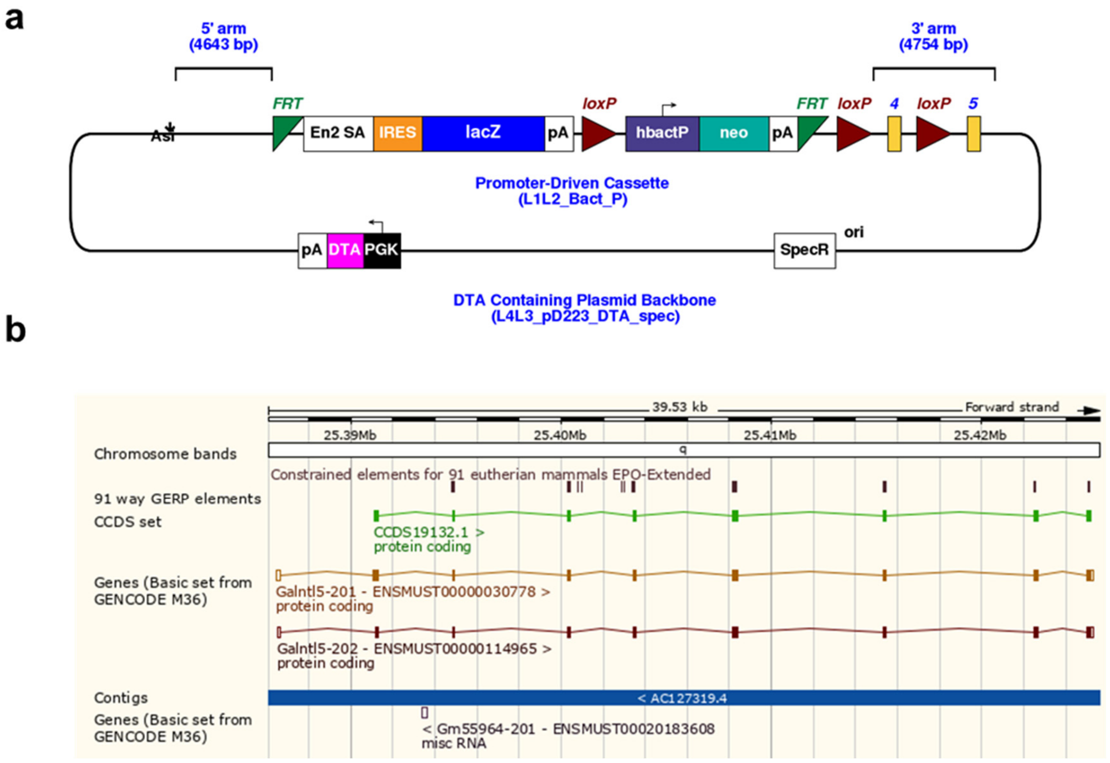

Supplementary Figure 7. A targeting vector design for the generation of *Galntl5* mutants and a sequence map of the genomic region encoding mouse *Galntl5*.

a) **A targeting vector to generate *Galntl5* mutant mice.** *Galntl5* mutant mice registered on the IMPC database were generated using this targeting vector, and then the promoter-driven neomycin resistance cassette and exon 4 of *Galntl5* were deleted by the Cre-loxP system.

b) **A sequence map of the genomic region encoding *Galntl5*.** *Galntl5* is transcribed into two splice variants. *Gm55964*, a non-coding RNA, exists in the intron sequence after exon 2 of *Galntl5*, a part of the sequence coding *Gm55964* was deleted in *Galntl5* mutant mice generated by Takasaki and colleagues[1].

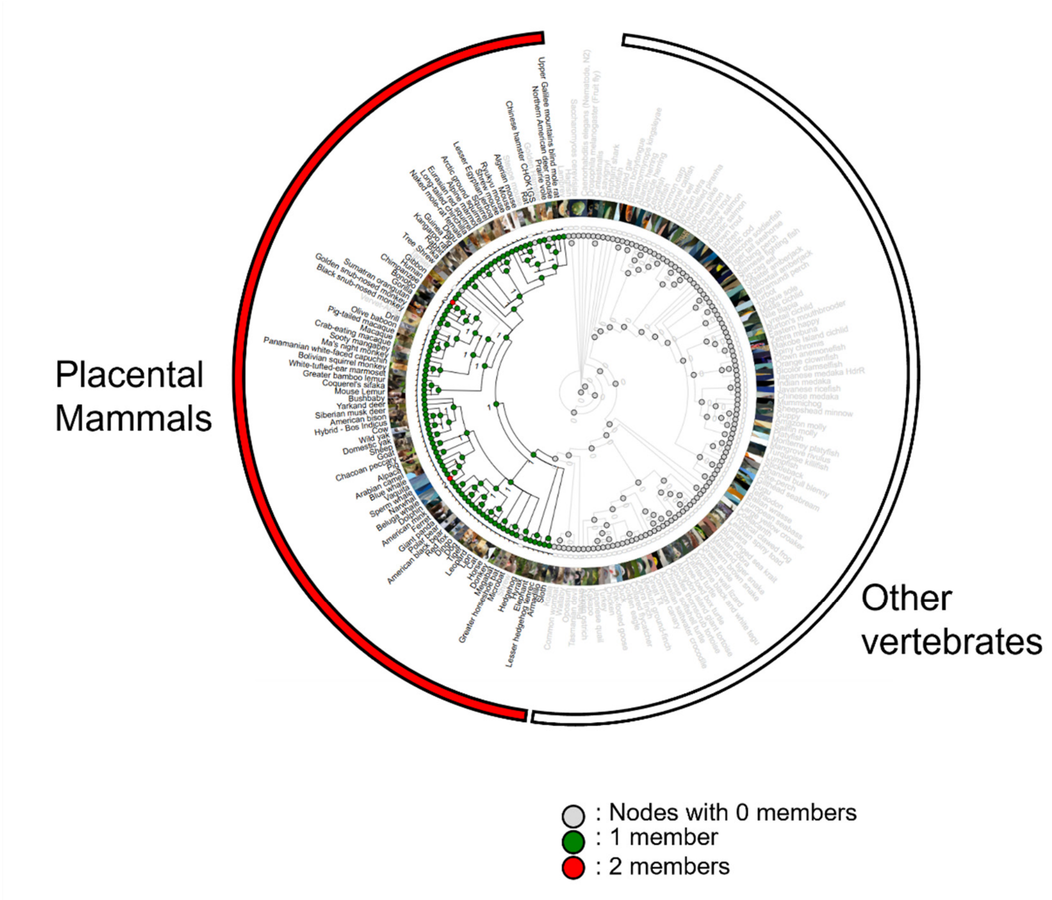

Supplementary Figure 8. Gene tree of *Galntl5*.

This tree was made based on the ensemble database.

Supplementary Table 1. Male fertility in each line of *Galntl5* mutant males

Figure #	Genotype	Pups/plug			
		Male #1	Male #2	Male #3	Male #4
1g	WT	7.2 (9 plugs)	8.1 (7 plugs)	9.1 (8 plugs)	
	Het	7.4 (9 plugs)	10.4 (5 plugs)	10.3 (6 plugs)	10.7 (7 plugs)
	em1 KO	0.8 (6 plugs)	0 (5 plugs)		
	em2 KO	0.3 (10 plugs)	0 (12 plugs)	0.2 (9 plugs)	
5d	Ctrl	7.8 (9 plugs)	6.6 (7 plugs)	7.4 (8 plugs)	
	em3 KO	0 (9 plugs)	0 (9 plugs)	0.7 (6 plugs)	
	em4 KO	0.3 (11 plugs)	0 (6 plugs)	0 (8 plugs)	

Supplementary Table 2. PCR primers

Figure	Sequence (5' to 3')	Name
1b, 1c	CTTCATACTCCCTTGCTCTCG (Fw4)	*Galntl5*
	CTGTGTTAAATGGCTCCAACTC (Rv4)	
	TGGATATGCCCTTGACTATAATGAG (Fw5)	*Hprt*
	TGGCAACATCAACAGGACTC (Rv5)	
1d	TTGGACAGTATGACAAGGATATGG (Fw6)	*GALNTL5*
	TGTTTCTTACTGATATGTCCTACTCG (Rv6)	
	AATCCCATCACCATCTTCCAG (Fw7)	*GAPDH*
	ATGACCCTTTTGGCTCCC (Rv7)	
S1b	TGAAACCCACAATGAAAAGTG (Fw3)	*Galntl5* (genotyping for *em1*)
	CATCACAATTTGTCCCTGGA (Rv1)	
S1b	GAGGAACTCCTAAAGAAGAG (Fw1)	*Galntl5* (genotyping for *em2* mutation)
	CATCACAATTTGTCCCTGGA (Rv1)	
	TGCTCAAGGGGTAAGGCAAG (Fw2)	
	CTCTGAGTGACGGGTTTGCT (Rv2)	
S1b	TGCTCAAGGGGTAAGGCAAG (Fw2)	*Galntl5* (genotyping for *em3* and *em4* mutation)
	CTCTGAGTGACGGGTTTGCT (Rv2)	
	CACTTTTCATTGTGGGTTTC (Rv3)	
S5a	AAAAGCTTGCCGCCATGAAAAGTGTCATAATTCA (Fw8)	An expression vector for ~ 50 kDa of mouse GALNTL5
	AAGAATTCGAAACGATTTTTTTTCCTTT (Rv8)	
S5a	AAAAGCTTGCCGCCATGCCATTCAATCTACCCAC (Fw9)	An expression vector for ~ 37 kDa of mouse GALNTL5
	CCTGAGGAGTCTCGAGCTAAGCGTAATCTGGAACG (Rv9)	
S5a	AAAAGCTTGCCGCCATGGAAATTTTTCGTGGAAAAGT (Fw10)	An expression vector for ~ 30 kDa of mouse GALNTL5
	AAGAATTCGAAACGATTTTTTTTCCTTT (Rv8)	
S5a	AAAAGCTTGCCGCCATGGCTTTTGACTGGAATCTGAA (Fw11)	An expression vector for ~ 20 kDa of mouse GALNTL5
	AAGAATTCGAAACGATTTTTTTTCCTTT (Rv8)	
S5a	AAAAGCTTGCCGCCATGCACAGGCGTGCAAACCAGAG (Fw12)	An expression vector for ~ 10 kDa of mouse GALNTL5
	AAGAATTCGAAACGATTTTTTTTCCTTT (Rv8)	
S5d	AATCTAGAGCCGCCATGAGAAATGCCATAATTCA (Fw13)	An expression vector for human GALNTL5
	AAGAATTCCAGGCTGTTCACAGATGCCT (Rv10)	

Supplementary Table 3. PCR conditions

Figure	Target	DNA polymerase	Primers	PCR condition: Initial denaturation	Denaturation	Annealing	Extension	cycle	Final extension	Predicted size
1b, 1c	*Galntl5*	Taq NEB	Fw4 + Rv4	95°C 2 min	95°C 30 sec	53°C 45 sec	68°C 30 sec	30 or 35	68°C 5 min	275 bp
1b, 1c	*Hprt*	Taq NEB	Fw5 + Rv5	95°C 2 min	95°C 30 sec	53°C 45 sec	68°C 30 sec	35	68°C 5 min	142 bp
1d	*GALNTL5*	Taq NEB	Fw6 + Rv6	95°C 2 min	95°C 30 sec	53°C 45 sec	68°C 30 sec	35	68°C 5 min	130 bp
1d	*GAPDH*	Taq NEB	Fw7 + Rv7	95°C 2 min	95°C 30 sec	53°C 45 sec	68°C 30 sec	30	68°C 5 min	146 bp
S1b	*Galntl5 em1* mutation	KOD-Fx neo	Fw3 + Rv1	94°C 3 min	94°C 30 sec	65°C 30 sec	72°C 30 sec	40	72°C 2 min	264 bp (*wt*), 247 bp (e*m1*)
S1b	*Galntl5 em2* mutation	KOD One	Fw1 + Rv1	98°C 3 min	98°C 10 sec	55°C 5 sec	72°C 1 sec	40	72°C 2 min	136 bp (*wt*)
			Fw2 + Rv2	98°C 3 min	98°C 10 sec	65°C 5 sec	72°C 1 sec	40	72°C 2 min	447 bp (*wt*), 422 bp (*em2*)
S1b	*Galntl5 em3* and *em4* mutation	KOD One	Fw2 + Rv2	98°C 3 min	98°C 10 sec	65°C 5 sec	72°C 1 sec	40	72°C 2 min	447 bp (*wt*), 442 bp (*em3*), 449 bp (*em4*)
			Fw2 + Rv3	98°C 3 min	98°C 10 sec	55°C 5 sec	72°C 1 sec	40	72°C 2 min	277 bp (*wt*)
S5a	An expression vector for ~ 50 kDa of mouse GALNTL5	KOD-Fx neo	Fw8 + Rv8	94°C 3 min	94°C 30 sec	65°C 30 sec	72°C 1.5 min	30	72°C 2 min	1,315 bp
S5a	An expression vector for ~ 37 kDa of mouse GALNTL5	KOD One	Fw9 + Rv9	98°C 3 min	98°C 10 sec	60°C 5 sec	72°C 10 sec	30	72°C 2 min	1,113 bp

S5a	An expression vector for ~ 30 kDa of mouse GALNTL5	KOD One	Fw10 + Rv8	98°C 3 min	98°C 10 sec	60°C 5 sec	72°C 10 sec	30	72°C 2 min	808 bp
S5a	An expression vector for ~ 20 kDa of mouse GALNTL5	KOD One	Fw11 + Rv8	98°C 3 min	98°C 10 sec	60°C 5 sec	72°C 1 sec	40	72°C 2 min	553 bp
S5a	An expression vector for ~ 10 kDa of mouse GALNTL5	KOD One	Fw12 + Rv8	98°C 3 min	98°C 10 sec	60°C 5 sec	72°C 1 sec	40	72°C 2 min	274 bp
S5d	An expression vector for human GALNTL5	KOD One	Fw13 + Rv10	98°C 3 min	98°C 10 sec	55°C 5 sec	72°C 10 sec	35	72°C 2 min	1,351 bp

Supplementary Table 4. Antibodies/lectins used in this study

Protein target or Antibodies	Company, Catalog#, Antigen sequence	Species raised in	References	Dilution used
A. Primary antibodies				
ADAM3	Santa Cruz Biotechnology, sc-365288	Mouse monoclonal IgG		1:1,000
Acetylated tubulin	Thermo Fisher, T7451	Mouse monoclonal IgG		1:1,000
BASIGIN (also known as EMMPRIN/CD147)	Custom antibody (pos2)	Rabbit polyclonal IgG		1:1,000
GALNTL5 (N)	Custom antibody, amino acids 34 to 52 (MLENEKEELLKKRSLGKNA) in UniProt accession# Q9D4M9	Rabbit polyclonal IgG	Generated in this study	1:500
GALNTL5 (C)	Custom antibody, amino acids 413 to 431 (IFPELEPFNTERKRKKNRF) in UniProt accession# Q9D4M9	Rabbit polyclonal IgG	Generated in this study	1:500-1:1,000
IZUMO1	Custom antibody (KS64-125) previously	Rat monoclonal IgG	PMID: 21131354[2]	1:1,000
LYPD4	Thermo Fisher Scientific, PA5-23848	Rabbit polyclonal IgG	PMID: 31455729[3]	1:1,000
LY6K	Kindly gifted by Dr. Yoshiko Araki	Rabbit polyclonal IgG	PMID: 24501175[4], PMID: 18503752[5]	1:1,000
	MBL, D363-3	Rat monoclonal IgG		1:500
tACE	Custom antibody (1D5)	Mouse monoclonal IgG	PMID: 16870943[6]	1:1,000
ZPBP1	Custom antibody (G176)	Goat polyclonal IgG	PMID: 17664285[7]	1:1,000
B. Secondary antibodies				
Anti-goat IgG-HRP	Jackson ImmunoResearch Laboratories, 805-035-180	Bovine		1:1,000
Anti-mouse IgG-HRP	Jackson ImmunoResearch Laboratories, 115-036-062	Goat		1:1,000
Anti-rabbit IgG-HRP	Jackson ImmunoResearch Laboratories, 111-036-045	Goat		1:1,000
Anti-rat IgG-HRP	Jackson ImmunoResearch Laboratories, 112-035-167	Goat		1:1,000

Streptavidin-HRP	Thermo Fisher Scientific, 21130 or N100	Streptomyces avidin	1:5,000
C. Lectins			
Con A-Biotinylated	Mitsubishi Gas Chemical Company, J203<br>Vector Laboratories, B-1005	Canavalia ensiformis<br>(jack bean)	1:1,000
Rhodamine-DBA	Vector Laboratories, RL-1032	Dolichos biflorus	1:100
GNL-Biotinylated	Vector Laboratories, GSK-2000	Galanthus Nivalis	1:100
GS-I or GS-I-biotinylated	EY Laboratories, B-2401-2 or BA-2401-2	Griffonia simplicifolia	1:1,000
LSL-N-Biotinylated	TCI, R0231	Laetiporus sulphureus	1:1,000
MAL-II-Biotinylated	Vector Laboratories, B-1265	Maackia amurensis<br>(Amur Maackia)	1:1,000
PNA-Biotinylated	Mitsubishi Gas Chemical Company, J214	Arachis hypogaea<br>(peanut)	1:1,000
WFA-Biotinylated	Vector Laboratories, GSK-2000	Wisteria Fluoribunda	1:100

Supplementary References

1 Takasaki, N. *et al.* A heterozygous mutation of GALNTL5 affects male infertility with impairment of sperm motility. *Proceedings of the National Academy of Sciences* **111**, 1120-1125 (2014).

2 Ikawa, M. *et al.* Calsperin is a testis-specific chaperone required for sperm fertility. *J Biol Chem* **286**, 5639-5646 (2011).

3 Fujihara, Y. *et al.* Identification of multiple male reproductive tract-specific proteins that regulate sperm migration through the oviduct in mice. *Proc Natl Acad Sci U S A* **116**, 18498-18506 (2019).

4 Fujihara, Y., Okabe, M. & Ikawa, M. GPI-anchored protein complex, LY6K/TEX101, is required for sperm migration into the oviduct and male fertility in mice. *Biol Reprod* **90**, 60 (2014).

5 Yoshitake, H. *et al.* TEX101, a germ cell-marker glycoprotein, is associated with lymphocyte antigen 6 complex locus k within the mouse testis. *Biochem Biophys Res Commun* **372**, 277-282 (2008).

6 Yamaguchi, R., Yamagata, K., Ikawa, M., Moss, S. B. & Okabe, M. Aberrant distribution of ADAM3 in sperm from both angiotensin-converting enzyme (Ace)- and calmegin (Clgn)-deficient mice. *Biol Reprod* **75**, 760-766 (2006).

7 Lin, Y. N., Roy, A., Yan, W., Burns, K. H. & Matzuk, M. M. Loss of zona pellucida binding proteins in the acrosomal matrix disrupts acrosome biogenesis and sperm morphogenesis. *Mol Cell Biol* **27**, 6794-6805 (2007).
